# Using HPLC-MS/MS to Determine the Loss of Primary and Secondary Metabolites in the Dehydration Process of Apple Slices

**DOI:** 10.3390/foods12061201

**Published:** 2023-03-12

**Authors:** Jan Juhart, Aljaz Medic, Jerneja Jakopic, Robert Veberic, Metka Hudina, Franci Stampar

**Affiliations:** Department of Agronomy, Biotechnical Faculty, University of Ljubljana, SI 1000 Ljubljana, Slovenia

**Keywords:** *Malus domestica* Borkh., organic acids, sugars, phenolic compounds, dehydrated apple slices, processing, HPLC-MS identification

## Abstract

The aim of this study was to compare peeled and unpeeled dehydrated apple slices of the red-fleshed ‘Baya Marisa’ and the white-fleshed ‘Golden Delicious’, to analyze the difference in the content of sugars, organic acids, and phenolic compounds during the heat process of dehydration, and to compare it with our previous study on fresh apples of the same cultivar. The purpose of these study was to see how many primary and secondary metabolites are lost in the dehydration process to better understand what is ingested by consumers in terms of nutritional value. A total of 30 phenolic compounds were identified and quantified, some of them for the first time. The total analyzed phenolic content (TAPC) of the unpeeled dehydrated apple slices was 1.7 times higher in ‘Golden Delicious’ than in ‘Baya Marisa’. The unpeeled dehydrated apple slices of ‘Golden Delicious’ had higher total hydroxycinnamic acid (2.7×) and dihydrochalcone (1.2×) content. The peeled dehydrated apple slices of ‘Baya Marisa’ had higher total dihydrochalcone (2.2×) and total flavanol (2.2×) content compared to ‘Golden Delicious’. The content of citric and malic acids was higher in the unpeeled and peeled dehydrated apple slices of ‘Baya Marisa’, compared to ‘Golden Delicious’. The content of ascorbic acid was higher in the unpeeled (1.6×) and peeled (1.8×) dried apple slices of ‘Baya Marisa’. The content of fructose and glucose was 1.4 times higher in the unpeeled dried apple slices of ‘Golden Delicious’.

## 1. Introduction

The color and flavor of apples are determined by organic acids, sugars, and phenolic compounds, which are influenced by cultivar, growing season, storage, and climatic conditions [1]. Apples (*Malus domestica* Borkh.) and their products contain substantial amounts of bioactive compounds, which are known to be beneficial for human health [2]. Five main groups of phenolics are found in apple fruits: anthocyanins, dihydrochalcones, hydroxycinnamic acids, flavanols, and flavonols, the content of which varies between different apple cultivars [3].

Red-skinned apples look tastier to consumers and usually contain higher amounts of phenolic compounds in the skin than green/yellow-skinned apples [4]. Anthocyanins are responsible for the red color of the skin and the flesh of some apple cultivars in which the *MdMYB10* gene is excessively expressed, which correlates with the expression of anthocyanin contents in the flesh of the apples [5]. Anthocyanins, particularly cyanidin and peonidin glycosides, are important for the red color of the flesh and skin of apples [6].

Studies on red-fleshed apples have shown that dehydrated apple slices and dried apples can be a healthy and good alternative to other fruit snacks because the preservation of phenolic compounds depends on the dehydration technique [7].

The most efficient and oldest food preservation method is dehydration, which removes free water to minimize microbial spoilage and increase shelf life [7]. Dehydrated apple fruit is commonly used for immediate consumption or as an ingredient in certain foods in many European countries. Therefore, there is a large market for dehydrated fruits, especially apples [7]. Hot air drying is the most commonly used process for dehydrating apple products because it is relatively inexpensive. However, this process can affect the quality of the products, such as color, texture, flavor, and metabolic profile [8].

The objective of this study was to compare in detail the differences in organic acid, sugar, and phenolic compound content of dehydrated (hot air process) red-fleshed and red-skinned apples of the cultivar ‘Baya Marisa’ and white-fleshed and yellow-peeled apples of the cultivar ‘Golden Delicious’, and to determine the durability or loss of sugars, organic acids, and especially phenolic compounds during the hot air drying. The purpose of this study was to see how many primary and secondary metabolites are lost in the dehydration process to better understand what is ingested by consumers in terms of nutritional value. This study is a continuation of our previous study comparing the ‘Baya Marisa’ with ‘Golden Delicious’, where ‘Baya Marisa’ had a higher content of dihydrochalcones, anthocyanins, and flavonols, while ‘Golden Delicious’ had higher levels of hydroxycinnamic acids and flavanols. The results of this study are important for future research on the effects of food dehydration on the metabolic profile of foods and, consequently, for human health and nutritionists, as it is one of the most prospective and detailed studies.

## 2. Materials and Methods

### 2.1. Plant Material

The two apple cultivars ‘Baya Marisa’ and ‘Golden Delicious’ were collected from intensive apple orchard in Zdole (45°9800200 N; 15°5200700 E; 307 m.a.s.l.) in the southeastern part of Slovenia. Apple trees of both cultivars were 5 years old at the time of sampling, grafted to rootstock M9, and spaced 3.2 m × 0.9 m apart. The rows were oriented in a north–south direction. Both apple cultivars, ‘Baya Marisa’ and ‘Golden Delicious’, were managed according to standard integrated pest management and harvested at their technological maturity. ‘Golden Delicious’ was harvested on 8 September 2022, and ‘Baya Marisa’ was harvested on 16 September 2022, and transferred to the laboratory for analysis and further processing.

**Figure 1:**
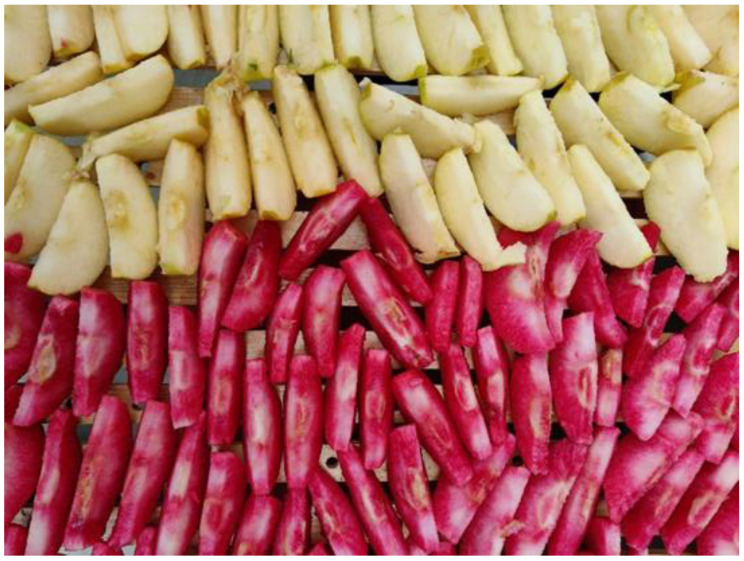
Apple slices of white-fleshed cultivar ‘Golden Delicious’ (above) and apple slices of red-fleshed cultivar ‘Baya Marisa’ (below), prepared for dehydration.

The analysis was performed with 18 apples per cultivar. The remaining 6 apples per cultivar (3 fruits of each cultivar were peeled and 3 were left unpeeled) were used for determining the dry matter. Extraction was performed in the laboratory of the Department of Agronomy, Biotechnical Faculty, University of Ljubljana (Ljubljana, Slovenia), where the analysis of organic acids, sugars, and phenolic compounds was also performed. Eighteen apples of each cultivar were randomly selected as replicates for metabolite analysis. The cores of the apples were removed and the apples were then sliced equally with a knife as shown in Figure 1. The skin was removed from nine apples of each cultivar while nine apples remained unpeeled and placed into a dehydrator. Apples were dehydrated using a standard air-drying method (24 h at 60 ± 1 °C, 15% relative humidity) in an air-convection oven [9].

### 2.2. Dry Matter

To determine dry matter (%), six apples per cultivar, ‘Baya Marisa’ and ‘Golden Delicious’, were obtained and cut into apple slices (3 fruits of each cultivar were peeled and 3 were unpeeled), weighed, and placed in the oven at 105 °C until the mass was constant.

### 2.3. Extraction of Organic Acids, Sugars, and Phenolic Compunds

Extraction of organic acids, sugars, and phenolic compounds was performed according to previous protocols [10,11,12], with some minor modifications. Eighteen apples of each cultivar (‘Baya Marisa’ and ‘Golden Delicious’) represented three biological replicates with six apple fruits each.

For extraction, dehydrated apple slices from each apple were frozen with liquid nitrogen and crushed in a mortar. Briefly, for the phenolic compounds, 1.0 g of crushed apple slices was extracted with 4 mL of 80% methanol (Sigma-Aldrich, Steiheim, Germany) containing 3% formic acid in bi-distilled water. The extraction ratio for the samples was 1:4 (*w/v*). Samples were then sonicated in ice-cold water for 60 min (Sonis 4 ultrasonic bath; Iskra pio, Sentjernej, Slovenia). Samples were then centrifuged at 4 °C for 10 min at 10,000 *g* (5810 R; Eppendorf, Hamburg, Germany), filtered through 0.2 µm polyamide filters (Chromafil AO −20/25; Macherey-Nagel, Düren, Germany), and stored in vials at −20 °C for further analysis.

The extraction of ascorbic acid was analogous to previous procedures [13]. Briefly, 1.0 g of the sample was extracted with 5 mL of 2% metaphosphoric acid in bi-distilled water, the extraction ratio being 1:5 (*w/v*).

The extraction ratio for organic acids and sugars was 1:5 (*w/v*); briefly, 1.0 g of the sample was mixed with 5 mL of bi-distilled water. Samples were then placed in tubes on a Unimax 1010 shaker (Heidolph Instruments, Schwabach, Germany) for 30 min. Centrifugation was then performed at 10,000× *g* for 10 min at 4 °C (5810 R; Eppendorf, Hamburg, Germany). For filtration, 0.2 µm cellulose filters (Chromafil Xtra MV −20/25; Macherey-Nagel, Düren, Germany) were used to fill the vials, which were then stored at −20 °C for further analysis.

### 2.4. HPLC Analysis of Organic Acids and Sugars

Individual sugars and organic acids were identified using Vanquish, a UHPLC (Thermo Scientific, San Jose, CA, USA), and commercial standards. The Rezex RCM monosaccharide column (Phenomenex, Torrance, CA, USA) operated at 65 °C was used to separate individual sugars and organic acids at a flow rate of 0.6 mL/min. Eluted carbohydrates were measured using a refractive index (RI) detector (Refractomax 520, Idex health and science KK 5-8-6, Nishiaoki Kawaguchi, Japan) according to the literature [14]. Organic acids were identified using a UV detector at 210 nm and a Rezex ROA column from Phenomenex at 65 °C on the same UHPLC system as described in the literature [11]. An amount of 4 mM sulfuric acid in bi-distilled water was used for organic acids and bi-distilled water for sugars. For ascorbic acid analysis, the same method was used as described in the literature [15]. The content of sugars and organic acids was expressed in mg/kg dehydrated weight (D).

### 2.5. HPLC-Mass Spectrometry Analysis for Phenolic Compounds

A UHPLC system (Thermo Scientific; San Jose, CA, USA) was used for the analysis of phenolic compounds. The diode array detector was set at 530 nm for anthocyanins, 350 nm for flavonols, and 280 nm for the other phenolic compounds. The conditions were as previously described in the protocol [10]. The injection volume was 20 µL, and the recorded spectra were from 200 nm to 600 nm. Compounds were separated using a Gemini 150 × 4.60 mm, 3 µm; C18 column (Phenomenex, Torrance, CA, USA) operated at 25 °C. Phenolic compounds were identified by tandem mass spectrometry (MS/MS; LCQ Deca XP MAX; Thermo Scientific, Waltham, MA, USA) with heated electrospray ionization in negative-ion mode for the detection of hydroxycinnamic acids, flavanols, flavonols, and dihydrochalcones and in positive-ion mode for the detection of anthocyanins. MS Scans from m/z 50 to 2000 were obtained for analysis. Data acquisition was performed using Xcalibur 2.2 software (Thermo Fischer Scientific Institute, Waltham, MA, USA). External standards were used for identification and quantification when available. Unknown compounds were identified using MS fragmentation and literature data. Quantification was performed according to a similar standard.

Total analyzed phenolic content (TAPC) represents the sum of all phenolic compounds identified. Individual phenolic compounds and TAPC are expressed in mg/kg dehydrated weight (D).

### 2.6. Chemicals

The bi-distilled water used for sample preparation and analysis was purified using the Milli-Q water purification system (Millipore, Bedford, MA, USA). Formic acid and acetonitrile were HPLC-MS grade (Fluka Chemie GmbH, Buchs, Switzerland). Metaphosphoric acid was purchased from Sigma-Aldrich (Steinheim, Germany).

The following standards were used for the analysis: caffeic acid, citric acid, cyanidin-3-O-galactoside, (-)epicatehin, ferulic acid, fructose, fumaric acid, glucose, malic acid, sorbitol, sucrose, phloridzin, p-coumaric acid, procyanidin B1, quercetin-3-O-glucoside, quercetin-3-O-galactoside, and quercetin-3-O-rhamnoside (Fluka Chemie GmbH, Buchs, Switzerland); chlorogenic acid, shikimic acid, and quercetin-3-O-rutinoside (Sigma-Aldrich Chemie GmbH, Steinheim, Germany); cyanidin-3-O-arabinoside and quercetin-3-O-arabinofuranoside (Apin Chemicals, Abingdon, UK).

### 2.7. Statistical Analysis

The data were collected in Microsoft Excel 2016, while further statistical analysis was performed using the R commander program. Three replicates per treatment were performed. The data presented are means ± standard errors (SE). To determine differences between data, one-way analysis of variance (ANOVA) with Tukey tests was used. The significance of differences was calculated at a 95% confidence level.

## 3. Results and Discussion

### 3.1. Dry Matter

The average dry matter of the unpeeled apple slices of the red-fleshed cultivar ‘Baya Marisa’ was 82.5% ± 0.2%. The peeled apple slices of ‘Baya Marisa’ had an average dry matter of 81.5% ± 0.2%. The average dry matter of unpeeled apple slices of the white-fleshed cultivar ‘Golden Delicious’ was 82.3% ± 0.1%. Peeled apple slices of ‘Golden Delicious’ had a dry matter of 81.4% ± 0.1. No results with significant differences were obtained. As reported by Palmer et al. [16], dry matter in apple fruit ranges from 71% to 82%. Dry matter concentration is responsible for biological processes that affect carbohydrate status, texture characteristics, fruit flavor, and consumer acceptance [16].

### 3.2. Content of Sugars and Organic Acids

Consumer acceptance is influenced by the acidity and sweetness of apple fruit, so sugars and organic acids were analyzed. We identified four different sugars and three organic acids in dehydrated apple slices. Table 1 shows the content of sugars and organic acids in the peeled and unpeeled dehydrated apple slices in the cultivar ‘Baya Marisa’ and ‘Golden Delicious’.

The content of glucose and fructose was higher in the unpeeled, dehydrated apple slices of ‘Golden Delicious’ than in the unpeeled, dehydrated apple slices of ‘Baya Marisa’. Dehydrated apple fruits are known to have higher levels of sugars and organic acids as they are dehydrated and therefore sugars and organic acids are more concentrated, as reported by Zhu et al. [17] and Owusu et al. [18]. The highest values for both cultivars were for fructose and the lowest for sorbitol. We suggest that the conversion and translocation of sugars, sorbitol to fructose, may be the reason for these results, since only a small fraction of fructose is converted into starch at the apple ripening stage [19].

Among the organic acids, malic acid showed the highest content, followed by citric acid and ascorbic acid (see Table 1). The peeled and unpeeled apple slices of red-fleshed ‘Baya Marisa’ had higher contents of all three detected organic acids. The content of all three organic acids was higher in the dehydrated apple slices than in the results of Juhart et al. [20], who studied the fresh apple fruit of ‘Golden Delicious’ and ‘Baya Marisa’. Comparable results were reported by Ghinea et al. [21], where dried apple chips had higher organic acid content as they become more concentrated with water loss.

The sugar–acid ratio in the unpeeled dehydrated apple slices of the cultivar ‘Baya Marisa’ was 3.7, which was lower than that of the unpeeled dried apple slices of ‘Golden Delicious’, which was 6.7. The sugar–acid ratio in the peeled dehydrated apple slices of ‘Baya Marisa’ was 4.8, compared to peeled dehydrated apple slices of ‘Golden Delicious’, which was 6.0. The sugar–acid ratio in the peeled and unpeeled dehydrated apple slices of both apple cultivars was lower than the results obtained by Juhart et al. [20], where the sugar–acid ratio in fresh apple fruit of cultivar ‘Baya Marisa’ was 11.1 and in fresh apple fruit of cultivar ‘Golden Delicious’ was 14.7. The sugar–acid ratio determines the quality and taste of the product, which means that both cultivars have a ratio below 20, indicating that both cultivars taste sour, as reported by Begic-Akagi et al. [22]. Harvesting at technological maturity could be the reason for the low sugar–acid ratio, as it increases with storage time due to starch degradation and the release of free sugar [23].

### 3.3. Identification of Individual Phenolic Compounds

A total of 30 phenolic compounds were identified in unpeeled and peeled apple slices based on mass spectra and the literature. Among the phenolic compounds, 2 anthocyanins, 2 dihydrochalcones, 17 hydroxycinnamic acids, 5 flavanols, and 4 flavonols were identified, some for the first time. The phenolic compounds were identified based on the m/z (mass-to-charge ratio) of the characteristic molecular and fragmentation ions. The phenolic compounds were tentatively identified based on their pseudomolecular ions (i.e., [M − H]^−^ and M^+^ ions) when standards for phenolic compounds were not available. When possible, a comparison was made with authentic standards.

The data for all identified phenolic compounds in peeled and unpeeled dehydrated apple slices of ‘Baya Marisa’ and ‘Golden Delicious’ are shown in Table 2.

### 3.4. Quantification of Individual Phenolic Compounds and Total Analyzed Phenolic Content

We identified 30 different phenolic compounds in peeled and unpeeled dehydrated apple slices, which is less compared to our previous study on fresh apples that identified 46 different phenolic compounds. We hypothesized that some phenolic compounds were degraded during the heat process of dehydration. As shown in Table 3, phenolic compounds were identified in both peeled and unpeeled dehydrated apple slices of red-fleshed ‘Baya Marisa’ and white-fleshed ‘Golden Delicious’. The predominant phenolic compounds were hydroxycinnamic acids, dihydrochalcones, flavonols, flavanols, and anthocyanins. Similar phenolic compounds in apple fruit were previously described by Tsao et al. [24], Chinnici et al. [25] and Juhart et al. [20].

The predominant hydroxycinnamic acid in both peeled and unpeeled dehydrated apple slices of ‘Baya Marisa’ and ‘Golden Delicious’ cultivars was chlorogenic acid with the highest content, as shown in Table 3. Similar results were reported by Wojdyło et al. [7] for dry apple slices, in which chlorogenic acid was higher than in fresh apples after drying at 70 °C for 2 h. In our study, the content of total hydroxycinnamic acids in the peeled dehydrated apple slices was similar, while the content of total hydroxycinnamic acid in the unpeeled dehydrated apple slices of ‘Golden Delicious’ was higher than in the unpeeled apple slices of ‘Baya Marisa’. In the study conducted by Salazar-Orbea et al. [26], the processing techniques (chopping, preheating to 92 °C for 5 min, hot pureeing, recirculation at 90 °C for 2 min, hot deaeration and pasteurization at 99 °C for 1 min) resulted in an increase from 38.6 to 46.3% in the total hydroxycinnamic acid content. As reported by Fernández-Jalao et al. [27], cell wall disruption could be the reason for the significant increase in total hydroxycinnamic acid content, and enzyme activation by thermal treatment promoting the release of hydroxycinnamic acids. The general trend in the literature is the degradation of hydroxycinnamic acids after processing at high temperatures. Some authors reported significant increases (205–925%) in hydroxycinnamic acid content in pasteurized apple juices (90 °C/14.8 min) in mild treatments [26].

In this study, two dihydrochalcones were identified (phloridzin and phloretin-2-O-xyloside). The total dihydrochalcone content was higher in the unpeeled dehydrated apple slices of ‘Golden Delicious’ than in the unpeeled dehydrated apple slices of ‘Baya Marisa’, whereas the total dihydrochalcone content was higher in the peeled apple slices of ‘Baya Marisa” in comparison with the peeled apple slices of ‘Golden Delicious’. The study conducted by Juhart et al. [20] showed a higher content of dihydrochalcones in the peel and a lower one in the flesh of fresh apple fruits of cultivars ‘Golden Delicious’ and ‘Baya Marisa’ cultivars, which was attributed to the activity of chalcone synthase, which increases with anthocyanin production during fruit ripening, as reported by Ju et al. [28]. Similar results were reported by Salazar-Orbea et al. [29], who found an increase in dihydrochalcone content in different heat treatments compared to fresh apples. Higher levels of dihydrochalcone could be caused by the formation of chalcones and the opening of pyrylium rings, which are the first steps of anthocyanin degradation, due to processing at higher temperatures, as reported by Ioannou et al. [30].

The flavonols detected in our study were quercetin glycosides. The total flavonol content was the same in the unpeeled dehydrate apple slices of ‘Baya Marisa’, compared to the unpeeled dehydrated apple slices of ‘Golden Delicious’. This was also similar for the peeled dehydrated apple slices of the studied cultivars. In our previous study, the total flavonol content was higher overall in the fresh fruit of the red-fleshed cultivar ‘Baya Marisa’ and the white-fleshed cultivar ‘Golden Delicious’ than in dehydrated peeled and unpeeled apple slices. As reported by Sonawane and Arya [31], flavonol degradation is temperature dependent, as flavonol degradation was higher when dried at 80 °C than when dried at 60 °C. As reported by Odriozola-Serrano et al. [32], enzymatic and non-enzymatic reactions are responsible for oxidative degradation, hydrolysis, or the precipitation of flavonols during storage and heat processing. Madrau et al. [33] suggest that the degradation of flavonols correlates with temperature, i.e., the higher the temperature, the lower the total flavonol content. However, peeling is an important preparatory step in food processing and analysis, and peeling has been reported to cause a loss of up to 48% of total flavonol content in some fruits [34].

Flavanols are the product of the flavonoid pathway [35]. Epicatechin derivatives, procyanidin trimer and procyanidin dimer 4, had the highest contents of flavanols. The total flavanol content was similar in the unpeeled dehydrated apple slices of ‘Baya Marisa’ and the unpeeled dehydrated apple slices of ‘Golden Delicious’. ‘Golden Delicious’ had lower total flavanol content in the peeled dehydrated apple slices than the peeled dehydrated apple slices of ‘Baya Marisa’. In our previous article, the results showed that the total flavanol content in the fresh fruits of ‘Golden Delicious’ and ‘Baya Marisa’ was lower overall compared to the dehydrated apple slices in this study. Similar results were reported by Alongi et al. [36], who found an increase (71–1800%) in flavanols upon mild (71.7 °C/0.4 min) and intensive (90 °C/14.8 min) pasteurization of apple juice. However, Salazar-Orbea et al. [29] reported a decrease in flavanol content after thermal treatments. It is well known that food processing under heat leads to chemical changes in flavanols, such as polymerization, degradation, stability, and epimerization, as reported by Liu et al. [37]. In this study, we hypothesize that higher temperatures could inactivate the enzymes responsible for the hydrolysis and oxidation of flavanols as reported by Madrau et al. [33]. Dehydration by heat is expected to produce two phenomena: chemical modification (cleavage and epimerization) and enzymatic degradation, as reported by Fernandes et al. [38]. However, 60 °C is not a very harsh dehydration process to destroy flavanols, and it is reported that mild heat treatments can cause the up-regulation of flavanols, especially procyanidins, which are higher in this study in peeled and unpeeled dehydrated apple slices than in our previous study using fresh fruit of the same cultivars, which is due to the formation of a (non)covalent bond between procyanidin terminal units and the cell wall, as reported by Liu et al. [37]. As reported by Bourvellec et al. [39], the better extractability of procyanidins due to cell wall degradation during heating could be the reason for the overall higher flavanol content of the dehydrated apple slices of both cultivars compared to our previous study with fresh fruits of the same cultivar.

Of the anthocyanins, only two (cyanidin-3-O-galactoside, cyanidin-3-O-arabinoside) were detected in the red-fleshed and red-skinned apple cultivar ‘Baya Marisa’. No anthocyanins were detected in the white-fleshed and yellow-skinned apple cultivar ‘Golden Delicious’, which was expected since anthocyanins are associated with a red color. The results obtained by Juhart et al. [20] showed a higher total anthocyanin content in fresh apples of the cultivar ‘Baya Marisa’. As reported by Salazar-Orbea et al. [26], the higher the temperature, the greater the degradation of anthocyanins, which is due to the inactivation of enzymes. As reported by Patras et al. [40], oxidation by thermal treatment is the main reason for the degradation of anthocyanins, as covalent bonds are cleaved. Oxygen is an important factor for the degradation of anthocyanins, as it accelerates the degradation of anthocyanins by the action of oxidizing enzymes or by direct oxidative mechanisms, as reported by Patras et al. [40].

**Figure 2:**
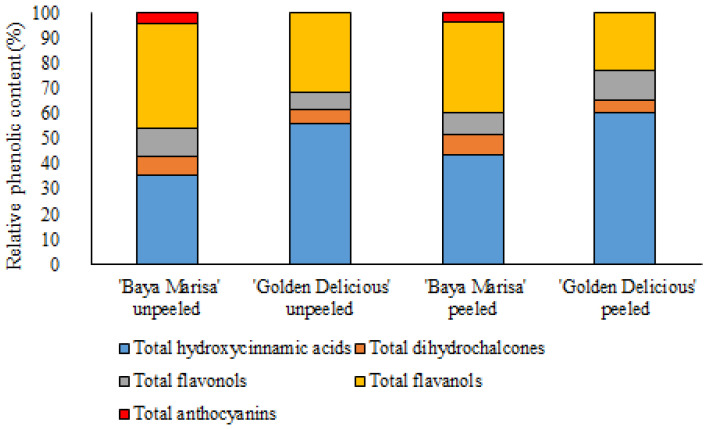
Relative content of identified phenolic compounds in unpeeled and peeled apple (*Malus domestica* Borkh.) slices of cultivars ‘Baya Marisa’ and ‘Golden Delicious’.

The TAPC value was higher in the unpeeled dehydrated apple slices of ‘Golden Delicious than in the unpeeled dehydrated apple slices of ‘Baya Marisa’. There were no statistically significant differences between the TAPC of the peeled dehydrated apple slices of ‘Baya Marisa’ and ‘Golden Delicious’. The total hydroxycinnamic acid content was higher in the unpeeled dehydrated apple slices of ‘Golden Delicious’, compared to ‘Baya Marisa’, while the total hydroxycinnamic acid content in the peeled dehydrated apple slices of ‘Golden Delicious’ and ‘Baya Marisa’ was similar. The total dihydrochalcone content was higher in the unpeeled dehydrated apple slices of ‘Golden Delicious’, compared to ‘Baya Marisa’, while total dihydrochalcone content was higher in the peeled dehydrated apple slices of ‘Baya Marisa’, compared to the peeled dehydrated apple slices of ‘Golden Delicious’. Total flavonol content was higher in the unpeeled dehydrated apple slices of both cultivars, compared to the peeled dehydrated apple slices of ‘Baya Marisa’ and ‘Golden Delicious’, while there were no statistically significant differences between the cultivars. Anthocyanins were detected only in ‘Baya Marisa’, as expected, since the flesh and peel of ‘Baya Marisa’ are red and that of ‘Golden Delicious’ are white/yellow. The most important phenolic groups were hydroxycinnamic acids and flavanols for both peeled and unpeeled dehydrated apple slices for both cultivars, as shown in Figure 2.

Overall, the TAPC in the dehydrated peeled apple slices of ‘Baya Marisa’ and ‘Golden Delicious’ was higher than the TAPC in the flesh of fresh fruits of the same cultivars in our previous study [20]. The TAPC in the skin of ‘Baya Marisa’ was higher than that in the unpeeled, dehydrated apple slices of ‘Baya Marisa’, while the TAPC in the dehydrated, unpeeled apple slices of ‘Golden Delicious’ was higher than that in the peel of the fresh fruit of ‘Golden Delicious’.

Our results show that peeled and unpeeled dehydrated apple slices had the same or higher levels of hydroxycinnamic acid, dihydrochalcone, and flavanols, while the levels of flavonols and anthocyanins were higher in the fresh fruit of the same cultivars.

## 4. Conclusions

This study found higher TAPC in dehydrated apple slices for both cultivars, and higher levels of sugars and organic acids because they are more concentrated during dehydration than in fresh apples of the same cultivars. For consumers, this means that for the same weight of consumption of fresh apples compared to dehydrated apple slices of both cultivars, the intake of total phenolic compounds is higher in dehydrated apple slices. This suggests that the dehydration of apple slices could be a good technique for food preservation, as dehydration prolongs the shelf life of products, increases their stability, and makes them microbiologically safe. Higher organic acid and sugar content suggests that quality characteristics such as taste, appearance, and color are preserved.

The apple cultivar ‘Baya Marisa’ is known to be acidic and astringent, which means that it is not usually consumed fresh, although our results in a previous study showed that it is rich in phenolic compounds. Due to the higher sugar and organic acid content resulting from dehydration, ‘Baya Marisa’ could be made more palatable by processing.

Unpeeled dehydrated apple slices of both cultivars have higher TAPC than peeled dehydrated apple slices, suggesting that phenolic compounds are mainly located in the skin of apples, as already observed in fresh apples; therefore, in terms of consumers, the unpeeled apple slices would be a recommended choice as they contain more phenolic compounds which are linked to have several health benefits.

We propose to perform further analyses for both apple cultivars on dehydrated apple slices and other processing methods with different processing techniques to determine the loss or increase in phenolic compounds, organic acids, and sugars with temperature, for the red-fleshed ‘Baya Marisa’ and the white-fleshed ‘Golden Delicious’.

## Figures and Tables

**Table 1:** Individual sugars and organic acids (mean ± SE, in g/kg D) in unpeeled and peeled dehydrated apple slices of red-fleshed cultivar ‘Baya Marisa’ and white-fleshed cultivar ‘Golden Delicious’.

	Unpeeled		Peeled	
Compunds	‘Baya Marisa’	‘Golden Delicious’	‘Baya Marisa’	‘Golden Delicious’
Sugars				
Sucrose	165.34 ± 4.41 a	159.40 ± 2.45 a	173.82 ± 5.53 a	173.65 ± 5.91 a
Glucose	74.26 ± 2.19 b	104.53 ± 5.90 a	79.46 ± 3.00 b	72.40 ± 1.19 b
Fructose	231.87 ± 5.53 b	319.66 ± 22.50 a	230.34 ± 7.93 b	242.50 ± 3.16 b
Sorbitol	26.11 ± 0.77 a	30.17 ± 1.32 a	26.81 ± 1.09 a	23.78 ± 0.19 a
Organic acids				
Citric acid	45.66 ± 0.57 a	26.04 ± 1.50 b	28.85 ± 1.28 b	20.38 ± 0.75 c
Malic acid	89.46 ± 2.23 a	65.73 ± 0.57 c	77.92 ± 0.36 b	64.95 ± 3.31 c
Ascorbic acid	0.09 ± 0.01 a	0.06 ± 0.00 b	0.08 ± 0.00 a	0.04 ± 0.00 b

Same letters between treatments following mean values are not significantly different (*p* < 0.05).

**Table 2:** Tentative identification of the 30 phenolic compounds of dehydrated apple slices of *Malus domestica* Borkh. cultivars ‘Baya Marisa’ and ‘Golden Delicious’ and used standards.

Phenolic Compounds	Rt (min)	[M − H]^−^ (m/z)	[M + H]^+^ (m/z)	MS^2^ (m/z)	MS^3^ (m/z)	Expressed as	Unpeeled		Peeled	
							‘Baya Marisa’	‘Golden Delicious’	‘Baya Marisa’	‘Golden Delicious’
Hydroxycinnamic acids										
Caffeic acid derivative	15.8	335		179,135		caffeic acid	X	X	X	X
Caffeic acid derivative 2	12.9	311		179		caffeic acid	X		X	
Dicaffeic acid derivative 1	10.8	457		179,135		caffeic acid	X		X	
Dicaffeic acid derivative 2	19.3	403		233,179,135		caffeic acid	X	X		X
Dicaffeic acid derivative 3	22.6	429		249,205,179,135		caffeic acid	X	X	X	X
Dicaffeic acid derivative 5	11.5	457		277,189,179	179,135	caffeic acid	X		X	
Dihydrodicaffeic acid derivative 2	20.4	405		225,181		caffeic acid	X	X	X	X
5-*O*-*p*-coumaroylquinic acid	16.5	337		191,163,119		chlorogenic acid	X	X	X	X
Chlorogenic acid (5-caffeoylquinic acid)	13.4	353		191,179		chlorogenic acid	X	X	X	X
Caffeoylferuoylquinic acid	14.2	563		385,205	191,193	chlorogenic acid				X
Caffeoylquinnic acid	14.1	353		191,179		chlorogenic acid		X		
Caffeoylquinnic acid derivative	12.4	451		353,311	191,179,135	chlorogenic acid		X		X
Feruloylquinic acid derivative 1	12.7	431		385,331	193,191	chlorogenic acid	X		X	
Caffeoylferuoylquinnic acid 1	13.9	563		385,321,205	193,191	ferulic acid	X	X	X	X
Feruoylquinnic acid hexoside	20.8	547		385,325	193,191	ferulic acid	X	X	X	X
Ferulic acid hexoside derivative	11.6	401		355	265,235,193	ferulic acid	X		X	
Cryptochlorogenic acid (4-caffeoylquinic acid)	14.7	353		191,179		cryptochlorogenic acid	X	X	X	X
Dihydrochalcones										
Phloridzin	23.9	481		435,273		phloridzin	X	X	X	X
Phloretin-2-*O*-xyloside	22.2	567		273,167		phloridzin	X	X	X	X
Flavonols										
Quercetin-3-*O*-arabinofuranoside	22.9	433		301,3		quercetin-3-*O*-arabinofuranoside	X	X	X	X
Quercetin-3-*O*-galactoside	21.2	463		301,3		quercetin-3-*O*-galactoside	X	X	X	X
Quercetin-3-*O*-glucoside	21.4	463		301,3		quercetin-3-*O*-glucoside	X	X	X	X
Quercetin-3-*O*-rhamnoside	23.0	447		301,3		quercetin-3-*O*-rhamnoside	X	X	X	X
**Flavanols**										
(-)epicatechin	15.8	289		271,245,205,179		(-) epicatechin		X		X
(Epi)catechin derivative	18.6	583		289,271	271,245,205179	(-) epicatechin	X	X	X	X
Flavanol monomer	22.0	289		245,205,179		(-) epicatechin	X		X	
Procyanidin dimer 4	14.6	577		451,425,407	289,245	procyanidin B1	X	X	X	X
Procyanidin trimer	16.9	865		739,695,577		procyanidin B1	X		X	
**Anthocyanins**										
Cyanidin-3-*O*-galactoside	8.9		449	287		cyanidin-3-*O*-galactoside	X		X	
Cyanidin-3-*O*-arabinoside	1.9		419	287		cyanidin-3-*O*-arabinoside	X		X	

Rt, retention time; [M − H]^−^, pseudomolecular ion identified in a negative ion mode; M^+^, pseudomolecular ion identified in a positive ion mode; x, presence of the identified compound.

**Table 3:** Individual and total analyzed phenolic compounds (TAPC) in dehydrated unpeeled and peeled apple slices of the two apple (*Malus domestica* Borkh.) cultivars ‘Baya Marisa’ and ‘Golden Delicious’ (mean ± SE, in mg/kg D).

	Unpeeled		Peeled	
Compounds	‘Baya Marisa’	‘Golden Delicious’	‘Baya Marisa’	‘Golden Delicious’
**Hydroxycinnamic acids**				
Caffeic acid derivative	9.6 ± 2.9 b	22.6 ± 1.0 a	8.5 ± 1.5 b	4.8 ± 0.6 b
Caffeic acid derivative 2	19.7 ± 3.9 a	nd	23.9 ± 1.3 a	nd
Dicaffeic acid derivative 1	5.8 ± 1.5 a	nd	5.4 ± 0.9 a	nd
Dicaffeic acid derivative 2	5.9 ± 1.8 a	5.4 ± 0.5 a	3.3 ± 1.0 ab	0.8 ± 0.05 b
Dicaffeic acid derivative 3	4.9 ± 1.1 ab	6.8 ± 0.4 a	3.0 ± 0.7 b	2.9 ± 0.5 b
Dicaffeic acid derivative 5	2.2 ± 0.7 a	nd	1.8 ± 0.2 a	nd
Dihydrodicaffeic acid derivative 2	8.9 ± 2.2 a	8.4 ± 0.7 a	5.0 ± 1.4 b	1.6 ± 0.3 c
5-*O*-*p*-coumaroylquinic acid	6.3 ± 1.0 c	80.9 ± 2.9 a	4.0 ± 0.9 c	23.5 ± 3.1 b
Chlorogenic acid (5-caffeoylquinic acid)	107.9 ± 19.5 c	377.1 ± 4.1 a	138.8 ± 5.6 bc	164.6 ± 11.7 b
Caffeoylferuoylquinic acid	nd	14.7 ± 1.6	nd	nd
Caffeoylquinic acid	nd	nd	nd	3.5 ± 1.1
Caffeoylquinic acid derivative	nd	139.8 ± 19.0 a	nd	23.3 ± 5.2 b
Feruloylquinnic acid derivative 1	19.6 ± 0.4 a	nd	19.2 ± 1.6 a	nd
Caffeoyl feruoylquinic acid 1	19.5 ± 1.3 b	36.4 ± 2.9 a	10.9 ± 1.8 c	9.6 ± 2.3 c
Ferulic acid hexoside derivative	3.9 ± 0.3 a	nd	2.6 ± 0.5 a	nd
Feruoylquinic acid hexoside	24.4 ± 1.7 a	16.3 ± 1.5 b	7.5 ± 0.9 c	7.2 ± 1.8 c
Cryptochlorogenic acid (4-caffeoylquinic acid)	31.9 ± 2.9 a	34.7 ± 2.3 a	19.6 ± 2.6 b	14.3 ± 0.8 b
Dihydrochalcones				
Phloridzin	35.3 ± 4.3 a	49.3 ± 0.9 a	20.5 ± 2.9 b	11.5 ± 2.3 c
Phloretin-2-*O*-xyloside	22.9 ± 3.7 a	23.2 ± 3.3 a	26.4 ± 2.0 a	10.1 ± 1.4 b
Flavonols				
Quercetin-3-*O*-arabinofuranoside	17.0 ± 2.7 a	9.5 ± 0.6 b	8.3 ± 1.3 b	8.0 ± 1.5 b
Quercetin-3-*O*-galactoside	11.4 ± 0.7 b	18.7 ± 1.6 a	6.2 ± 1.8 c	8.2 ± 1.3 c
Quercetin-3-*O*-glucoside	23.6 ± 2.0 a	32.6 ± 0.9 a	14.5 ± 3.1 b	15.8 ± 3.1 b
Quercetin-3-*O*-rhamnoside	30.3 ± 1.2 a	30.6 ± 1.0 a	20.2 ± 3.2 b	18.7 ± 2.8 b
Flavanols				
(-)epicatechin	nd	126.2 ± 2.9 a	nd	21.2 ± 5.6 b
Epicatechin derivative	78.6 ± 5.3 a	56.0 ± 0.6 ab	43.2 ± 6.9 b	27.2 ± 4.4 c
Flavanol monomer	8.8 ± 0.6 a	nd	1.8 ± 0.5 b	nd
Procyanidin dimer 4	116.2 ± 33.3 b	237.2 ± 33.7 a	86.7 ± 16.3 bc	48.3 ± 14.5 c
Procyanidin trimer	111.4 ± 6.4 a	nd	78.5 ± 9.7 b	nd
Anthocyanins				
Cyanidin-3-galactoside	6.8 ± 1.6 a	nd	3.4 ± 0.2 b	nd
Cyanidin-3-arabinoside	27.5 ± 3.0 a	nd	15.8 ± 1.8 b	nd
Total hydroxycinnamic acids	270.6 ± 42.5 b	743.1 ± 34.5 a	253.6 ± 19.9 b	256.0 ± 27.1 b
Total dihydrochalcones	58.2 ± 8.0 b	72.5 ± 2.5 a	46.9 ± 4.9 b	21.5 ± 3.6 c
Total flavonols	82.3 ± 6.5 a	91.3 ± 4.2 a	49.3 ± 9.5 b	50.7 ± 7.8 b
Total flavanols	315.0 ± 44.6 ab	419.4 ± 36.1 a	210.0 ± 31.4 b	96.8 ± 24.3 c
Total anthocyanins	34.3 ± 2.9 a	nd	19.2 ± 2.0 b	nd
TAPC	760.4 ± 103.5 b	1326.4 ± 72.4 a	579.2 ± 66.2 b	425.0 ± 62.0 b

Presented data are means ± standard error. Means followed by different letters in unpeeled or peeled dehydrated apple slices, respectively (within rows), are significantly different (*p* < 0.05); nd, not detected.

## Data Availability

The data are available from the corresponding author.
